# Complete Genome Sequence of *Dyella* sp. Strain GSA-30, a Predominant Endophytic Bacterium of *Dendrobium* Plants

**DOI:** 10.1128/mra.01338-22

**Published:** 2023-03-06

**Authors:** Tomoki Nishioka, Kana Morinaga, Hideyuki Tamaki

**Affiliations:** a Bioproduction Research Institute, National Institute of Advanced and Industrial Science and Technology, Tsukuba, Ibaraki, Japan; b Faculty of Life and Environmental Sciences, University of Tsukuba, Tsukuba, Ibaraki, Japan; c Microbiology Research Center for Sustainability, University of Tsukuba, Tsukuba, Ibaraki, Japan; d Biotechnology Research Center, The University of Tokyo, Tokyo, Japan; University of Arizona

## Abstract

We report a complete genome sequence of *Dyella* sp. strain GSA-30, a predominant endophytic bacterium of *Dendrobium* plants. The genome consists of a circular 5,501,810-bp chromosome with a G+C content of 61.4%. The genome was predicted to harbor 6 rRNA genes, 51 tRNA genes, and 4,713 coding sequences.

## ANNOUNCEMENT

A recent 16S rRNA gene sequencing survey revealed that the genus *Dyella* is one of the predominant bacterial taxa inhabiting the inside *Dendrobium* roots (1). Although *Dendrobium* plants are orchids with ornamental and medicinal values, many of them are threatened with extinction (2, 3). *Dyella* spp. may play a key role in the growth of *Dendrobium* plants, because they have been reported to possess plant growth-promoting traits such as indole acetic acid production, aminocyclopropane carboxylic acid production, β-1,3-glucanase production, and phosphate solubilization (4, 5). We previously isolated various endophytic bacteria of green stem *Dendrobium* roots using diluted tryptic soy medium prepared by autoclaving phosphate and agar separately (1). Based on the 16S rRNA gene sequencing analysis, one of the isolates, designated strain GSA-30, was classified into genus *Dyella*. Here, we provide the complete genome sequence of *Dyella* sp. strain GSA-30.

A single colony of strain GSA-30 was incubated in 1/5-strength tryptic soy broth (Becton, Dickinson and Company, Sparks, MD, USA) at 25°C for 24 h, with shaking at 120 rpm. The cultured cells were harvested by centrifugation and then lysed with lysozyme and achromopeptidase (6). Genomic DNA was extracted using the phenol-chloroform method as described previously (7). The genomic DNA obtained was subjected to the following sequencing analyses. *De novo* sequencing was conducted using the MiSeq sequencing platform (Illumina, San Diego, CA, USA) and the GridION X5 system (Oxford Nanopore Technologies [ONT], Oxford, UK). For MiSeq sequencing, a sequencing library was prepared using the Nextera DNA Flex library preparation kit (Illumina), and paired-end (2 × 256-bp) reads were obtained. Default parameters were used for all software unless otherwise specified. The raw sequencing data (862,322 reads [average read length, 247.3 bp]) were processed using fastp v.0.20.1 (8) to trim adapters and select high-quality data (Phred quality scores of >30), which resulted in 711,048 reads with an average length of 241.6 bp. For GridION X5 sequencing, a sequencing library was constructed using a ligation sequencing kit (SQK-LSK110; ONT) without shearing. The prepared library was applied to an R9.4.1 flow cell (FLO-MIN106; ONT). GridION X5 sequencing yielded a total of 370,362 reads, with an *N*_50_ value of 4,053 bp. Quality control using Filtlong v.0.2.1 (https://github.com/rrwick/Filtlong) selected sequence reads that were >3,000 bp long with a >90% identity match to the Illumina short reads, which generated a total of 86,368 reads (696 Mb), with an *N*_50_ value of 9,850 bp.

For complete *de novo* genome assembly, the processed short and long reads were coassembled using the Unicycler v.0.4.8 pipeline (9). The presence of contaminant DNA was assessed using BlobTools v.1.0 (10). The contig sequence obtained was polished using Pilon v.1.24 (11). CheckM v.1.2.2 was used to check the quality of the assembled genome sequence, with completeness of 99.8% and contamination of 2.0% (12). Automatic annotation was performed using the annotation pipeline DFAST v.1.2.18 (13). The complete *Dyella* sp. strain GSA-30 genome comprises a circular 5,501,810-bp chromosome and exhibits a G+C content of 61.4%, with 4,713 coding sequences, 6 rRNA genes, and 51 tRNA genes.

### Data availability.

The complete genome sequence and annotations for *Dyella* sp. strain GSA-30 have been deposited in DDBJ/EMBL/GenBank under accession number AP027042. The genome sequence has also been submitted to the SRA under BioSample accession number SAMD00557612 and BioProject accession number PRJDB14759. Raw sequencing data for strain GSA-30 were deposited under DRA accession numbers DRR420783 (Illumina) and DRR420784 (Nanopore).
